# Supplementary material for: A Likelihood Approach for Real-Time Calibration of Stochastic Compartmental Epidemic Models
Source: PLoS Comput Biol. 2017 Jan 17;13(1):e1005257. doi: 10.1371/journal.pcbi.1005257 (PMC5240920; doi:10.1371/journal.pcbi.1005257)
Supplement: S1 File — (TAR.GZ) [file pcbi.1005257.s014.tar.gz › HSPH_Online-SI-Revision/IPoi09/n100-severe/n100-severe_BENCH2_table4.pdf]

1	newly 3 weeks spec	1675.	3934.25	{2155., 6135.}	134.97	0.326715	newly 3 weeks cumul	7166.	14 378.5	{8469., 22 198.}	100.925	0.272622	newly 5 weeks spec	875.	2519.92	{1223., 3964.}	187.991	0.396921	newly 5 weeks cumul	9240.	20 073.7	{11 377., 31 243.}	117.341	0.299794	newly 46 weeks cumul	11 305.	28 557.5	{15 152., 44 737.}	152.609	0.354866
2	newly 3 weeks spec	260.	1096.21	{440., 1543.}	321.619	0.516387	newly 3 weeks cumul	1782.	5811.47	{2323., 8475.}	226.495	0.417784	newly 5 weeks spec	65.	391.339	{157., 598.}	502.06	0.665813	newly 5 weeks cumul	1949.	6857.74	{2759., 10 043.}	251.972	0.447599	newly 53 weeks cumul	2047.	7421.24	{3027., 10 828.}	262.575	0.459199
3	newly 3 weeks spec	220.	914.836	{461., 1580.}	315.835	0.55171	newly 3 weeks cumul	1526.	4955.9	{2498., 8915.}	224.764	0.447251	newly 5 weeks spec	41.	323.574	{167., 653.}	689.204	0.826297	newly 5 weeks cumul	1670.	5829.28	{2928., 10 575.}	249.059	0.477882	newly 54 weeks cumul	1741.	6312.52	{3165., 11 524.}	262.58	0.493972
4	newly 3 weeks spec	1102.	1530.61	{802., 2936.}	54.7626	0.159674	newly 3 weeks cumul	5564.	7028.31	{3862., 13 159.}	47.9543	0.154888	newly 5 weeks spec	463.	694.145	{368., 1348.}	61.05	0.166659	newly 5 weeks cumul	6701.	8759.44	{4787., 16 351.}	50.0592	0.155872	newly 52 weeks cumul	7579.	10 176.5	{5531., 19 043.}	51.924	0.157175
5	newly 3 weeks spec	2014.	2371.75	{1221., 4091.}	46.9753	0.16278	newly 3 weeks cumul	7895.	9152.95	{4878., 15 768.}	43.3773	0.155427	newly 5 weeks spec	1193.	1414.28	{694., 2424.}	50.6369	0.171922	newly 5 weeks cumul	10 658.	12 408.1	{6534., 21 376.}	44.8808	0.158811	newly 46 weeks cumul	14 294.	16 915.6	{8742., 29 117.}	47.2308	0.162926
6	newly 3 weeks spec	1306.	2121.43	{1081., 3251.}	72.8404	0.197843	newly 3 weeks cumul	6189.	9507.66	{5113., 14 687.}	64.9555	0.186409	newly 5 weeks spec	562.	994.951	{486., 1502.}	84.7383	0.213846	newly 5 weeks cumul	7603.	11 965.5	{6402., 18 280.}	68.0036	0.190639	newly 52 weeks cumul	8616.	14 050.9	{7416., 21 312.}	72.1595	0.196302
7	newly 3 weeks spec	678.	1908.73	{928., 2812.}	181.524	0.389629	newly 3 weeks cumul	3914.	9185.62	{4820., 13 310.}	134.947	0.319282	newly 5 weeks spec	239.	792.583	{416., 1161.}	231.625	0.456422	newly 5 weeks cumul	4550.	11 209.4	{5897., 16 273.}	146.391	0.337528	newly 53 weeks cumul	4820.	12 600.7	{6608., 18 278.}	161.424	0.361815
8	newly 3 weeks spec	1197.	3296.2	{1631., 5855.}	175.371	0.383406	newly 3 weeks cumul	5691.	13 019.9	{6877., 22 875.}	129.127	0.317255	newly 5 weeks spec	560.	1849.28	{886., 3385.}	230.228	0.447961	newly 5 weeks cumul	7047.	17 349.5	{9020., 30 864.}	146.396	0.343074	newly 49 weeks cumul	8398.	22 342.4	{11 249., 39 833.}	166.066	0.370098
9	newly 3 weeks spec	121.	2292.08	{1133., 3777.}	1794.28	1.20844	newly 3 weeks cumul	1328.	10 953.	{5904., 18 350.}	724.77	0.857597	newly 5 weeks spec	4.	957.371	{493., 1724.}	23 834.3	2.30331	newly 5 weeks cumul	1373.	13 399.2	{7126., 22 494.}	875.907	0.927913	newly 54 weeks cumul	1373.	15 066.9	{7942., 25 201.}	997.373	0.977028
10	newly 3 weeks spec	840.	3768.23	{2085., 6516.}	348.599	0.604023	newly 3 weeks cumul	4139.	13 957.7	{8450., 23 409.}	237.223	0.494697	newly 5 weeks spec	240.	2371.75	{1221., 4091.}	888.231	0.928722	newly 5 weeks cumul	4837.	19 342.4	{11 285., 32 661.}	299.884	0.561952	newly 47 weeks cumul	5228.	27 105.1	{15 043., 46 010.}	418.46	0.664764
11	newly 3 weeks spec	467.	1660.37	{907., 2701.}	255.54	0.492499	newly 3 weeks cumul	2925.	7966.92	{4375., 12 777.}	172.373	0.383008	newly 5 weeks spec	130.	690.968	{350., 1087.}	431.514	0.662122	newly 5 weeks cumul	3288.	9737.81	{5322., 15 571.}	196.162	0.417751	newly 53 weeks cumul	3458.	10 983.4	{6028., 17 567.}	217.623	0.446931
12	newly 3 weeks spec	1748.	3934.25	{2155., 6135.}	125.294	0.308796	newly 3 weeks cumul	7573.	14 378.5	{8469., 22 198.}	90.6016	0.250741	newly 5 weeks spec	887.	2519.92	{1223., 3964.}	184.094	0.391005	newly 5 weeks cumul	9747.	20 073.7	{11 377., 31 243.}	106.307	0.277801	newly 46 weeks cumul	12 152.	28 557.5	{15 152., 44 737.}	135.005	0.323498
13	newly 3 weeks spec	1801.	3175.34	{1631., 5081.}	80.6647	0.213345	newly 3 weeks cumul	7188.	11 889.9	{6617., 18 631.}	70.0158	0.198892	newly 5 weeks spec	954.	1972.13	{927., 3181.}	109.595	0.260416	newly 5 weeks cumul	9478.	16 381.9	{8709., 25 776.}	77.0848	0.210394	newly 46 weeks cumul	11 854.	22 893.5	{11 627., 36 089.}	95.6368	0.241899
14	newly 3 weeks spec	2332.	3156.93	{1767., 4751.}	48.5234	0.151467	newly 3 weeks cumul	8946.	11 641.9	{7045., 17 731.}	41.6447	0.138079	newly 5 weeks spec	1422.	2089.08	{1025., 2983.}	62.4508	0.170746	newly 5 weeks cumul	12 296.	16 298.2	{9419., 24 631.}	45.4207	0.146513	newly 46 weeks cumul	16 703.	23 931.2	{12 623., 34 398.}	56.2533	0.161505
15	newly 3 weeks spec	957.	3560.21	{1749., 6178.}	272.018	0.513735	newly 3 weeks cumul	4781.	13 410.5	{7192., 22 968.}	180.495	0.406394	newly 5 weeks spec	410.	2133.58	{971., 3735.}	420.386	0.642002	newly 5 weeks cumul	5823.	18 321.4	{9446., 31 581.}	214.638	0.449456	newly 48 weeks cumul	6577.	24 748.6	{12 212., 42 911.}	276.29	0.518165
16	newly 3 weeks spec	2249.	3934.25	{2155., 6135.}	78.4349	0.215186	newly 3 weeks cumul	9354.	14 378.5	{8469., 22 198.}	58.5968	0.179288	newly 5 weeks spec	1205.	2519.92	{1223., 3964.}	110.754	0.2656	newly 5 weeks cumul	12 257.	20 073.7	{11 377., 31 243.}	67.9114	0.196572	newly 47 weeks cumul	15 533.	28 557.5	{15 152., 44 737.}	86.4946	0.229355
17	newly 3 weeks spec	539.	2621.36	{1303., 4344.}	386.338	0.62783	newly 3 weeks cumul	2954.	12 042.2	{6255., 19 609.}	307.657	0.560874	newly 5 weeks spec	198.	1155.72	{551., 1811.}	483.698	0.702585	newly 5 weeks cumul	3479.	14 947.4	{7623., 24 248.}	329.647	0.581162	newly 53 weeks cumul	3686.	17 141.7	{8657., 27 870.}	365.048	0.613855
18	newly 3 weeks spec	789.	2583.05	{1455., 4283.}	227.383	0.463241	newly 3 weeks cumul	4078.	11 269.3	{6536., 18 507.}	176.345	0.396615	newly 5 weeks spec	352.	1258.05	{679., 2053.}	257.4	0.493771	newly 5 weeks cumul	4968.	14 343.	{8258., 23 505.}	188.708	0.413187	newly 51 weeks cumul	5544.	17 101.6	{9745., 28 038.}	208.47	0.439866
19	newly 3 weeks spec	457.	1647.7	{740., 2561.}	260.548	0.482216	newly 3 weeks cumul	2528.	7577.38	{3526., 11 924.}	199.738	0.408789	newly 5 weeks spec	174.	739.279	{324., 1139.}	324.873	0.546734	newly 5 weeks cumul	2969.	9434.11	{4349., 14 712.}	217.754	0.43189	newly 51 weeks cumul	3262.	10 911.1	{5012., 16 933.}	234.492	0.452613
20	newly 3 weeks spec	674.	3296.2	{1631., 5855.}	389.05	0.63284	newly 3 weeks cumul	3591.	13 019.9	{6877., 22 875.}	262.57	0.515639	newly 5 weeks spec	266.	1849.28	{886., 3385.}	595.218	0.771267	newly 5 weeks cumul	4252.	17 349.5	{9020., 30 864.}	308.031	0.561593	newly 49 weeks cumul	4608.	22 342.4	{11 249., 39 833.}	384.862	0.63067
21	newly 3 weeks spec	521.	1231.23	{657., 1802.}	136.999	0.314098	newly 3 weeks cumul	3210.	6052.08	{3132., 8873.}	91.7066	0.232953	newly 5 weeks spec	157.	504.05	{271., 756.}	221.051	0.43976	newly 5 weeks cumul	3675.	7348.72	{3819., 10 717.}	102.425	0.253707	newly 53 weeks cumul	3774.	8235.88	{4259., 12 039.}	119.45	0.284731
22	newly 3 weeks spec	558.	2390.5	{1112., 4287.}	328.405	0.558875	newly 3 weeks cumul	3543.	9997.02	{4914., 18 145.}	182.162	0.392424	newly 5 weeks spec	178.	1225.8	{550., 2281.}	588.652	0.755093	newly 5 weeks cumul	4026.	12 945.3	{6197., 23 543.}	221.542	0.444465	newly 51 weeks cumul	4274.	15 889.	{7440., 28 889.}	271.76	0.502735
23	newly 3 weeks spec	35.	613.7	{288., 861.}	1653.43	1.16222	newly 3 weeks cumul	468.	3676.2	{1812., 5233.}	685.513	0.818542	newly 5 weeks spec	5.	184.433	{88., 281.}	3588.67	1.48326	newly 5 weeks cumul	490.	4198.63	{2074., 5999.}	756.864	0.855548	newly 55 weeks cumul	497.	4424.72	{2173., 6314.}	790.285	0.872116
24	newly 3 weeks spec	330.	859.694	{440., 1346.}	160.533	0.353563	newly 3 weeks cumul	2438.	5131.4	{2753., 8287.}	111.525	0.269187	newly 5 weeks spec	72.	261.129	{139., 396.}	262.679	0.494343	newly 5 weeks cumul	2664.	5867.1	{3138., 9344.}	120.921	0.286572	newly 56 weeks cumul	2695.	6193.19	{3311., 9790.}	130.174	0.303408
25	newly 3 weeks spec	192.	697.383	{322., 1122.}	263.22	0.493835	newly 3 weeks cumul	1247.	4382.03	{2098., 6767.}	251.406	0.483759	newly 5 weeks spec	31.	194.433	{94., 328.}	527.204	0.732475	newly 5 weeks cumul	1365.	4941.8	{2388., 7657.}	262.037	0.496473	newly 56 weeks cumul	1367.	5159.15	{2486., 7990.}	277.407	0.514434
26	newly 3 weeks spec	830.	3810.	{2008., 7154.}	359.036	0.610192	newly 3 weeks cumul	4614.	15 044.5	{8533., 26 935.}	226.063	0.474497	newly 5 weeks spec	289.	2120.26	{1032., 3850.}	633.655	0.801075	newly 5 weeks cumul	5390.	20 021.8	{10 897., 35 920.}	271.462	0.526058	newly 50 weeks cumul	5805.	25 585.4	{13 583., 46 206.}	340.747	0.595179
27	newly 3 weeks spec	578.	2790.27	{1386., 4886.}	382.746	0.618962	newly 3 weeks cumul	3242.	11 788.1	{6176., 20 608.}	263.607	0.507375	newly 5 weeks spec	170.	1408.15	{643., 2519.}	728.321	0.843126	newly 5 weeks cumul	3757.	15 186.7	{7751., 26 736.}	304.223	0.549409	newly 51 weeks cumul	4031.	18 435.6	{9169., 32 655.}	357.345	0.599654
28	newly 3 weeks spec	1075.	2082.8	{753., 3728.}	105.693	0.247474	newly 3 weeks cumul	4677.	9047.95	{3607., 16 619.}	102.384	0.2533	newly 5 weeks spec	430.	986.949	{348., 1739.}	137.864	0.279178	newly 5 weeks cumul	5845.	11 483.4	{4442., 20 835.}	105.792	0.254583	newly 50 weeks cumul	7034.	13 476.1	{5038., 24 152.}	102.582	0.24616
29	newly 3 weeks spec	909.	2790.27	{1386., 4886.}	206.961	0.422326	newly 3 weeks cumul	4464.	11 788.1	{6176., 20 608.}	164.074	0.368476	newly 5 weeks spec	364.	1408.15	{643., 2519.}	286.853	0.512474	newly 5 weeks cumul	5336.	15 186.7	{7751., 26 736.}	184.608	0.397035	newly 51 weeks cumul	5817.	18 435.6	{9169., 32 655.}	216.926	0.440368
30	newly 3 weeks spec	1036.	3768.23	{2085., 6516.}	263.729	0.512943	newly 3 weeks cumul	4857.	13 957.7	{8450., 23 409.}	187.372	0.425225	newly 5 weeks spec	473.	2371.75	{1221., 4091.}	401.428	0.634072	newly 5 weeks cumul	6087.	19 342.4	{11 285., 32 661.}	217.766	0.462125	newly 47 weeks cumul	7069.	27 105.1	{15 043., 46 010.}	283.436	0.533741
31	newly 3 weeks spec	35.	227.085	{110., 331.}	548.814	0.744747	newly 3 weeks cumul	499.	1608.2	{769., 2301.}	222.285	0.443101	newly 5 weeks spec	17.	54.2712	{23., 84.}	219.84	0.426544	newly 5 weeks cumul	538.	1775.17	{849., 2537.}	229.957	0.453171	newly 56 weeks cumul	540.	1827.27	{875., 2630.}	238.384	0.463983
32	newly 3 weeks spec	307.	1514.08	{798., 2846.}	393.186	0.638692	newly 3 weeks cumul	2113.	7717.21	{4136., 14 687.}	265.225	0.51295	newly 5 weeks spec	67.	586.148	{297., 1128.}	774.847	0.88398	newly 5 weeks cumul	2352.	9243.59	{4904., 17 744.}	293.01	0.543622	newly 54 weeks cumul	2475.	10 182.3	{5411., 19 615.}	311.406	0.562944
33	newly 3 weeks spec	386.	914.836	{461., 1580.}	137.208	0.308448	newly 3 weeks cumul	2219.	4955.9	{2498., 8915.}	124.325	0.28914	newly 5 weeks spec	106.	323.574	{167., 653.}	205.258	0.413775	newly 5 weeks cumul	2533.	5829.28	{2928., 10 575.}	130.66	0.299338	newly 54 weeks cumul	2695.	6312.52	{3165., 11 524.}	134.63	0.306005
34	newly 3 weeks spec	1133.	3668.27	{2096., 5612.}	223.766	0.469894	newly 3 weeks cumul	5334.	13 540.2	{8418., 20 819.}	153.847	0.377519	newly 5 weeks spec	504.	2386.57	{1296., 3732.}	373.525	0.612135	newly 5 weeks cumul	6608.	18 896.3	{11 420., 29 168.}	185.96	0.422165	newly 46 weeks cumul	7621.	26 953.6	{15 392., 41 027.}	253.675	0.502134
35	newly 3 weeks spec	193.	1054.66	{571., 1583.}	446.454	0.678006	newly 3 weeks cumul	1207.	5843.8	{3218., 8950.}	384.159	0.628918	newly 5 weeks spec	21.	362.148	{188., 602.}	1624.51	1.17337	newly 5 weeks cumul	1310.	6822.89	{3729., 10 448.}	420.831	0.659674	newly 55 weeks cumul	1313.	7312.57	{3962., 11 228.}	456.936	0.68837
36	newly 3 weeks spec	618.	2525.82	{1352., 4296.}	308.709	0.557233	newly 3 weeks cumul	3733.	11 502.8	{6481., 19 327.}	208.138	0.444709	newly 5 weeks spec	195.	1143.95	{613., 1947.}	486.642	0.707995	newly 5 weeks cumul	4250.	14 352.	{7922., 24 013.}	237.693	0.481845	newly 53 weeks cumul	4446.	16 578.9	{9033., 27 586.}	272.895	0.522916
37	newly 3 weeks spec	114.	624.032	{278., 1167.}	447.396	0.64807	newly 3 weeks cumul	920.	3891.52	{1669., 7429.}	322.992	0.541063	newly 5 weeks spec	26.	176.063	{81., 330.}	577.167	0.736249	newly 5 weeks cumul	1039.	4395.73	{1854., 8337.}	323.073	0.540252	newly 55 weeks cumul	1048.	4599.21	{1958., 8679.}	338.856	0.555981
38	newly 3 weeks spec	477.	1660.37	{907., 2701.}	248.086	0.483298	newly 3 weeks cumul	2807.	7966.92	{4375., 12 777.}	183.823	0.400892	newly 5 weeks spec	136.	690.968	{350., 1087.}	408.065	0.642527	newly 5 weeks cumul	3197.	9737.81	{5322., 15 571.}	204.592	0.42994	newly 53 weeks cumul	3359.	10 983.4	{6028., 17 567.}	226.984	0.459545
39	newly 3 weeks spec	862.	1459.16	{628., 2942.}	84.5929	0.227596	newly 3 weeks cumul	4078.	6659.59	{2929., 13 259.}	79.0331	0.220005	newly 5 weeks spec	352.	669.294	{267., 1366.}	101.303	0.249164	newly 5 weeks cumul	4972.	8323.32	{3604., 16 629.}	82.3926	0.224613	newly 50 weeks cumul	5595.	9695.85	{4114., 19 226.}	86.8964	0.230689
40	newly 3 weeks spec	607.	1845.44	{583., 3406.}	208.734	0.357246	newly 3 weeks cumul	3292.	8898.42	{3133., 17 268.}	175.147	0.34281	newly 5 weeks spec	210.	711.109	{180., 1278.}	243.835	0.377082	newly 5 weeks cumul	3890.	10 759.5	{3677., 20 710.}	181.729	0.345244	newly 53 weeks cumul	4289.	11 819.1	{3977., 22 716.}	181.125	0.340775
41	newly 3 weeks spec	231.	601.533	{283., 768.}	160.404	0.346205	newly 3 weeks cumul	1656.	3621.32	{1721., 4700.}	119.214	0.275915	newly 5 weeks spec	65.	182.183	{89., 230.}	180.282	0.377272	newly 5 weeks cumul	1848.	4139.32	{1963., 5396.}	124.319	0.285007	newly 55 weeks cumul	1919.	4367.7	{2091., 5738.}	127.796	0.291279
42	newly 3 weeks spec	1150.	3952.47	{1992., 7191.}	243.693	0.48549	newly 3 weeks cumul	5514.	14 759.7	{8092., 25 834.}	167.677	0.391935	newly 5 weeks spec	480.	2425.32	{1130., 4177.}	405.276	0.633919	newly 5 weeks cumul	6798.	20 303.1	{10 702., 35 448.}	198.663	0.432824	newly 48 weeks cumul	7662.	27 887.9	{13 963., 48 359.}	263.977	0.50912
43	newly 3 weeks spec	1025.	3320.19	{1733., 6074.}	223.921	0.453413	newly 3 weeks cumul	4521.	12 648.6	{7419., 22 360.}	179.775	0.407119	newly 5 weeks spec	563.	1974.58	{920., 3408.}	250.725	0.459483	newly 5 weeks cumul	5852.	17 197.1	{9645., 30 376.}	193.867	0.42003	newly 47 weeks cumul	6825.	22 880.4	{12 118., 40 184.}	235.243	0.464958
44	newly 3 weeks spec	487.	2292.08	{1133., 3777.}	370.653	0.603692	newly 3 weeks cumul	3260.	10 953.	{5904., 18 350.}	235.98	0.467577	newly 5 weeks spec	138.	957.371	{493., 1724.}	593.747	0.765495	newly 5 weeks cumul	3654.	13 399.2	{7126., 22 494.}	266.7	0.502815	newly 54 weeks cumul	3820.	15 066.9	{7942., 25 201.}	294.422	0.532635
45	newly 3 weeks spec	960.	2422.57	{1124., 3822.}	152.351	0.330732	newly 3 weeks cumul	5111.	10 858.8	{5342., 16 918.}	113.677	0.273682	newly 5 weeks spec	424.	1117.46	{495., 1703.}	163.977	0.343569	newly 5 weeks cumul	6116.	13 624.	{6576., 21 182.}	123.62	0.289286	newly 52 weeks cumul	6740.	15 840.2	{7541., 24 488.}	135.372	0.307692
46	newly 3 weeks spec	833.	3249.76	{1575., 5849.}	290.127	0.531977	newly 3 weeks cumul	4606.	13 300.9	{7179., 24 096.}	188.773	0.417284	newly 5 weeks spec	254.	1722.65	{784., 3117.}	578.207	0.755944	newly 5 weeks cumul	5399.	17 417.3	{9131., 31 500.}	222.602	0.459309	newly 51 weeks cumul	5711.	21 602.	{11 053., 39 127.}	278.252	0.522425
47	newly 3 weeks spec	1599.	3168.06	{1521., 5657.}	100.765	0.248401	newly 3 weeks cumul	6760.	12 101.	{6083., 21 264.}	82.4969	0.221105	newly 5 weeks spec	834.	1898.29	{847., 3551.}	129.191	0.288568	newly 5 weeks cumul	8781.	16 466.9	{8024., 28 968.}	90.5933	0.233329	newly 47 weeks cumul	10 888.	22 328.6	{10 452., 40 039.}	107.13	0.2595
48	newly 3 weeks spec	1207.	2422.57	{1124., 3822.}	104.353	0.248498	newly 3 weeks cumul	5943.	10 858.8	{5342., 16 918.}	87.4022	0.225055	newly 5 weeks spec	486.	1117.46	{495., 1703.}	131.974	0.292006	newly 5 weeks cumul	7196.	13 624.	{6576., 21 182.}	93.5809	0.235037	newly 52 weeks cumul	7925.	15 840.2	{7541., 24 488.}	102.962	0.250286
49	newly 3 weeks spec	149.	859.694	{440., 1346.}	476.976	0.698805	newly 3 weeks cumul	1122.	5131.4	{2753., 8287.}	357.344	0.601327	newly 5 weeks spec	19.	261.129	{139., 396.}	1274.36	1.07292	newly 5 weeks cumul	1199.	5867.1	{3138., 9344.}	389.333	0.630145	newly 56 weeks cumul	1212.	6193.19	{3311., 9790.}	410.99	0.648802
50	newly 3 weeks spec	163.	613.7	{288., 861.}	276.503	0.494105	newly 3 weeks cumul	1147.	3676.2	{1812., 5233.}	220.506	0.429225	newly 5 weeks spec	19.	184.433	{88., 281.}	870.702	0.903477	newly 5 weeks cumul	1229.	4198.63	{2074., 5999.}	241.63	0.456192	newly 55 weeks cumul	1262.	4424.72	{2173., 6314.}	250.611	0.467413
